# Exposure to Per- and Polyfluoroalkyl Substances and Timing of Puberty in Norwegian Boys: Data from the Bergen Growth Study 2

**DOI:** 10.1021/acs.est.4c06062

**Published:** 2024-09-03

**Authors:** Ingvild Halsør Forthun, Mathieu Roelants, Helle Katrine Knutsen, Line Småstuen Haug, Nina Iszatt, Lawrence M. Schell, Astanand Jugessur, Robert Bjerknes, Ninnie B. Oehme, Andre Madsen, Ingvild Særvold Bruserud, Petur Benedikt Juliusson

**Affiliations:** † Department of Clinical Science, University of Bergen, 5020 Bergen, Norway; ‡ Children and Youth Clinic, Haukeland University Hospital, 5021 Bergen, Norway; § Department of Public Health and Primary Care, Centre for Environment and Health KU Leuven, 3000 Leuven, Belgium; ∥ Department of Food Safety, Norwegian Institute of Public Health, 0213 Oslo, Norway; ⊥ Center for Sustainable Diets, Norwegian Institute of Public Health, 0213 Oslo, Norway; # Department of Epidemiology and Biostatistics, University at Albany, Albany, New York 12144, United States; ∇ Centre for Fertility and Health, Norwegian Institute of Public Health, 0213 Oslo, Norway; ○ Department of Global Public Health and Primary Care, University of Bergen, 5020 Bergen, Norway; ◆ Medical Biochemistry and Pharmacology, Haukeland University Hospital, 5021 Bergen, Norway; ¶ Department of Health Registry Research and Development, Norwegian Institute of Public Health, 5808 Bergen, Norway

**Keywords:** child, adolescent, endocrine disruption, environmental health, puberty

## Abstract

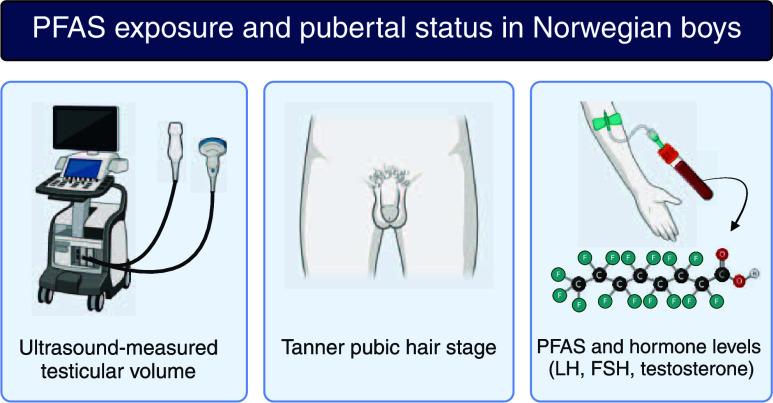

Per- and polyfluoroalkyl substances (PFAS) are widespread environmental contaminants with endocrine-disruptive properties. Their impact on puberty in boys is unclear. In this cross-sectional study, we investigated the association between PFAS exposure and pubertal timing in 300 Norwegian boys (9–16 years), enrolled in the Bergen Growth Study 2 during 2016. We measured 19 PFAS in serum samples and used objective pubertal markers, including ultrasound-measured testicular volume (USTV), Tanner staging of pubic hair development, and serum levels of testosterone, luteinizing hormone, and follicle-stimulating hormone. In addition to logistic regression of single pollutants and the sum of PFAS, Bayesian and elastic net regression were used to estimate the contribution of the individual PFAS. Higher levels of the sum of perfluorooctanesulfonic acid (PFOS), perfluorooctanoic acid (PFOA), perfluorononanoic acid (PFNA), and perfluorohexanesulfonic acid (PFHxS) were associated with later pubertal onset according to USTV (age-adjusted odds ratio (AOR): 2.20, 95% confidence interval (CI): 1.29, 3.93) and testosterone level (AOR: 2.35, 95% CI: 1.34, 4.36). Bayesian modeling showed that higher levels of PFNA and PFHxS were associated with later pubertal onset by USTV, while higher levels of PFNA and perfluoroundecanoic acid (PFUnDA) were associated with later pubertal onset by testosterone level. Our findings indicate that certain PFAS were associated with delay in male pubertal onset.

## Abstract 2

The impact of PFAS exposure on puberty is unclear. We found that higher PFAS concentrations were associated with later onset of objective pubertal markers in boys including ultrasound-measured testicular volume.

## Introduction

Per- and polyfluoroalkyl substances (PFAS) are a group of synthetic chemicals with a unique ability to repel water, oil, and dirt. These properties have led to their widespread use in a variety of industrial and consumer products.^1^ The chemical structure of PFAS, characterized by a strong carbon–fluorine bond, accounts for the environmental persistence and bioaccumulation potential of several PFAS.^2^ Humans are primarily exposed through ingestion of contaminated food and drinking water.^3−5^ Notably, PFAS are present at detectable levels in the blood of almost every individual, including children and teenagers,^6^ which documents the extensive spread and persistence of these contaminants.^3,7^

Exposure to PFAS has been associated with alterations in the endocrine system, including changes in pubertal timing and development.^8,9^ Rodent studies have found that both pre- and postnatal exposure to perfluorooctanoic acid (PFOA) are linked to delayed vaginal opening and impaired mammary gland maturation in the female offspring^10,11^ and delayed pubertal onset in male offspring measured as a delay in preputial separation (the separation of the prepuce from the glans penis).^12^

Only a few previous studies have investigated the association between PFAS concentrations in children’s blood and their pubertal development, showing varied results.^13−15^ However, several studies have shown associations between prenatal PFAS exposure and delayed pubertal development, particularly later menarche in girls.^16,17^ A systematic review of pre- and postnatal PFAS exposure and pubertal development concluded that data are still limited and inconsistent, primarily focused on girls and self-reported pubertal status.^18,19^

The purpose of the present study was to investigate whether PFAS serum concentrations were associated with later puberty in boys using several markers of pubertal development, including objective ultrasound measures of testicular volume (USTV), Tanner pubic hair (PH) stages, and serum hormone levels. To our knowledge, no previous study has used ultrasound to measure testicular volume to assess associations with PFAS exposure. This method can provide a more detailed insight into the potential influence of PFAS on the onset and progression of puberty, as ultrasound measurements are more precise and objective compared to self-reported pubertal status and orchidometer measurements.^20^

## Material and Methods

### Childhood Population

**Figure 1:**
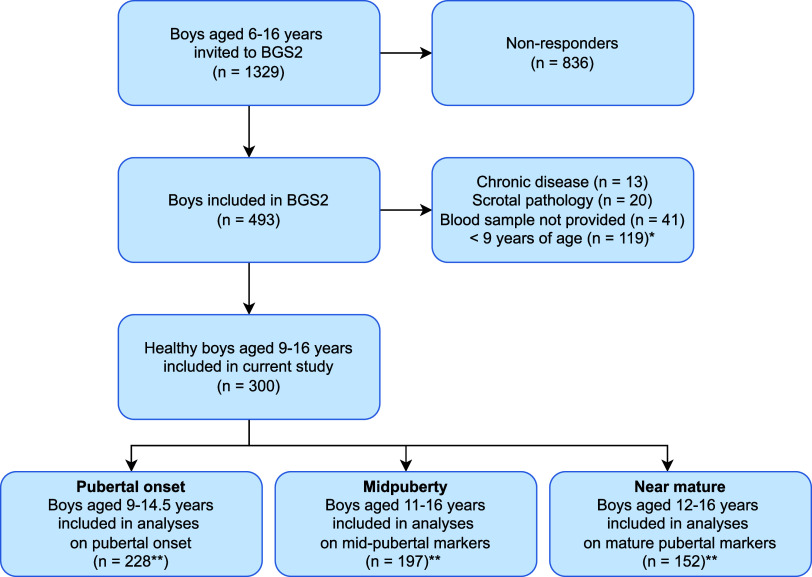
Flowchart of boys included in current study BGS2 = Bergen Growth Study 2. *To avoid inclusion of strictly prepubertal boys. **Due to missing data, certain individuals were excluded from specific analyses: Ultrasound-measured testicular volume (*n* = 6), Tanner pubic hair stage (*n* = 7), hormones (*n* = 7).

All children from six randomly selected public schools in Bergen, Norway, were invited to participate in the Bergen Growth Study 2 (BGS2), a cross-sectional study on puberty and growth conducted in January-June 2016.^21^ Of those invited, 493 boys between 6 and 16 years enrolled in the study, corresponding to a 37% participation rate. Out of these, 33 boys were excluded due to a chronic disease or scrotal pathology, and 41 did not provide a blood sample. In addition, boys below the lower age boundary for normal pubertal onset (<9 years of age) were excluded^22^ as they were all prepubertal based on their testicular volume. This resulted in a final cohort of 300 healthy boys aged 9 to 16 years (Figure 1). Within this group, six boys lacked data on testicular volume, seven were missing information on Tanner PH stage, and seven lacked data on testosterone, luteinizing hormone (LH), and/or follicle-stimulating hormone (FSH).

Analyses of pubertal onset were confined to boys aged between 9 and 14.5 years (*n* = 228) to exclude both strictly prepubertal and pubertal boys, while analyses concerning the midpubertal, and mature pubertal markers were based on boys aged 11–16 years (*n* = 197) and 12–16 years (*n* = 152), respectively. Examinations were conducted at school during school hours, while blood samples were collected on a separate day within 5 weeks of the examination, with an average interval of 11 days. A parental questionnaire was distributed to all participating boys, with a completion rate of 69.0%. A description of the study population (healthy boys aged 9–16 years, and prepubertal and pubertal boys aged 9–14.5 years) is presented in Table 1.

**Table 1:** Description of the Study Population (9–16 Years) and Boys Categorized as Prepubertal or Pubertal by Ultrasound-Measured Testicular Volume (9–14.5 Years)^a^

	**all boys(*n* = 300)**	**prepubertal boysUSTV < 2.7 mL(*n* = 131)**	**pubertal boysUSTV ≥ 2.7 mL(*n* = 93)**
**age (median)**	12.1 (4.06)	10.2 (1.46)	12.7 (1.87)
missing (*n*)	0	0	0
**BMI (median)**	17.9 (3.53)	16.5 (2.66)	18.7 (2.88)
missing (*n*)	3 (1.0%)	0	0
**parental educational level (*n*)**			
less than college/university	43 (14.3%)	15 (11.5%)	17 (18.3%)
college/university ≤4 years	58 (19.3%)	29 (22.1%)	15 (16.1%)
college/university >4 years	106 (35.3%)	54 (41.2%)	34 (36.6%)
missing	93 (31.0%)	33 (25.2%)	27 (29.0%)
**breastfeeding (*n*)**			
<6 months	41 (13.7%)	12 (9.2%)	18 (19.4%)
6–12 months	80 (26.7%)	37 (28.2%)	33 (35.5%)
>12 months	57 (19.0%)	36 (27.5%)	10 (10.8%)
missing	122 (40.7%)	46 (35.1%)	32 (34.4%)
**parental origin (*n*)**			
Norwegian	76 (25.3%)	30 (22.9%)	20 (21.5%)
European	14 (4.7%)	6 (4.6%)	5 (5.4%)
non-European	11 (3.7%)	6 (4.6%)	4 (4.3%)
missing	199 (66.3%)	89 (67.9%)	64 (68.8%)
**USTV (*n*)**			
<2.7 mL	163 (54.3%)	131 (100%)	0
≥2.7 mL	132 (44.0%)	0	93 (100%)
≥7.2 mL	98 (32.7%)	0	34 (36.6%)
≥17.6 mL	22 (7.3%)	0	1 (1.1%)
missing	6 (2.0%)	0	0
**Tanner PH stage (*n*)**			
1	132 (44.0%)	110 (84.0%)	21 (22.6%)
2	33 (11.0%)	13 (9.9%)	19 (20.4%)
3	29 (9.7%)	6 (4.6%)	23 (24.7%)
4	28 (9.3%)	0	13 (14.0%)
5	71 (23.7%)	0	16 (17.2%)
missing	7 (2.3%)	2 (1.5%)	1 (1.1%)
**testosterone level**			
**≥ 0.5 nmol/L (*n*)**			
yes	167 (55.7%)	9 (6.9%)	87 (93.5%)
no	130 (43.3%)	122 (93.1%)	6 (6.5%)
**testosterone (median)**	1.11 (10.3)	0.36 (0.95)	6.01 (5.71)
missing	3 (1.0%)	0	0
**LH (median)**	1.0 (2.0)	0.35 (0.48)	1.89 (1.35)
**LH *z*-score (mean)**	–0.04 (1.02)	–0.41 (1.00)	0.54 (0.78)
missing (*n*)	6 (2.0%)	1 (0.8%)	0
**FSH (median)**	2.1 (2.3)	1.48 (0.96)	3.08 (1.83)
**FSH *z*-score (mean)**	0.01 (1.01)	–0.09 (1.04)	0.21 (0.93)
missing (*n*)	3 (1.0%)	0	0

a USTV = ultrasound-measured testicular volume; Tanner PH = Tanner pubic hair stage. LH = luteinizing hormone in IU/L; FSH = follicle-stimulating hormone in IU/L. Testosterone is measured in nmol/L. Medians are presented with interquartile range, means with standard deviation, and numbers with percentages.

### Pubertal Development and Testicular Volume

Ultrasound examination of the right testicle was performed by a trained radiographer using a Sonosite Edge ultrasound machine with a 15–6 MHz linear probe, according to a standardized protocol.^23^ The ultrasound measurement had a technical error of measurement of 6.5%, while the intraobserver variability was 9.2%. The Lambert equation, TV = length × width × depth × 0.71,^24^ was used to calculate testicular volume. The equivalent Prader orchidometer volume was empirically derived from the ultrasound volume using Vol_OM_ = 1.96 × Vol_US_^0.71^. A Prader orchidometer volume of ≥4 mL marks the onset of puberty in boys and corresponds to an USTV of ≥2.7 mL.^23^ Further, a Prader orchidometer volume of 8 mL, which marks the midpubertal state, corresponds to an USTV of 7.2 mL. Finally, a Prader orchidometer volume of ≥15 mL, a mature testicular volume, corresponds to an USTV of ≥17.6 mL. Tanner PH was assessed by healthcare professionals based on the descriptions of Marshall and Tanner.^25^ Pubarche, the onset of pubic hair development, is defined as Tanner stage PH 2, while Tanner PH 5 is the mature stage.

### Biomarkers

Serum samples were collected at school by an experienced biomedical laboratory scientist between 08:00 AM and 2:00 PM. Nineteen PFAS, namely perfluorobutanoic acid (PFBA), perfluoropentanoic acid (PFPeA), perfluorohexanoic acid (PFHxA), perfluoroheptanoic acid (PFHpA), PFOA, perfluorononanoic acid (PFNA), perfluorodecanoic acid (PFDA), perfluoroundecanoic acid (PFUnDA), perfluorododecanoic acid (PFDoDA), perfluorotridecanoic acid (PFTrDA), perfluorotetradecanoic acid (PFTeDA), perfluorobutanesulfonic acid (PFBS), perfluorohexanesulfonic acid (PFHxS), perfluoroheptanesulfonic acid (PFHpS), perfluorooctanesulfonic acid (PFOS), perfluorodecanesulfonic acid (PFDS), perfluorooctanesulfonamide (PFOSA), *N*-methylperfluorooctanesulfonamide (MeFOSA) and N-ethylperfluorooctanesulfonamide (EtFOSA), were analyzed at the Norwegian Institute of Public Health, Oslo, Norway, using high-performance tandem mass spectrometry (LC-MS/MS), as described by Haug et al.^26^ The quality control of the assays is described by Forthun et al.^27^ The sum of PFOS, PFOA, PFNA and PFHxS (∑4PFAS), which accounted for approximately 95% of the total serum PFAS concentration in our sample, served as an estimate for overall PFAS exposure. ∑4PFAS has previously been used to assess health risks associated with PFAS exposure.^3^ We also analyzed associations with a weighted sum of near total PFAS exposure using relative potency factors that were based on blood concentrations and liver effects in male rats.^28^ Based on this study, the sum of PFOS, PFOA, PFNA and PFHxS, weighted by potency factors of 3, 1, 5, and 0.6, respectively, is referred to as the potency score.

Total testosterone was quantified at the Hormone Laboratory at Haukeland University Hospital in Bergen, Norway, by LC-MS/MS, following the method and quality control procedures described by Methlie et al.^29^ A testosterone concentration of 0.5 nmol/L or more was used as an alternative indicator of puberty onset.^30^ The Hormone Laboratory also analyzed LH and FSH using the IMMULITE 2000 XP platform (Siemens Healthcare). LH and FSH rise significantly with increasing pubertal stage.^31^ We calculated age-adjusted *z*-scores for male LH and FSH levels due to the pronounced age-dependent variation exhibited by these hormones.^32^

### Statistical Analysis

For our primary analyses, we investigated the odds of being in a given pubertal state according to PFAS exposure for three different age ranges: 9–14.5 years of age, 11–16 years of age, and 12–16 years of age. For this, we estimated age-adjusted odds ratio (AOR) of: (i) having a prepubertal testicular volume (USTV < 2.7 mL), (ii) having an USTV < 7.2 mL, and (iii) having an USTV < 17.6 mL, respectively. We then investigated the odds of: (i) prepubarche (Tanner PH stage <2) in boys aged 9–14.5 years, and (ii) having a Tanner PH stage less than 5 in boys aged 12–16 years. The referent group was Tanner PH ≥ 2 for boys 9–14.5 years, and Tanner PH 5 for boys aged 12–16 years. Finally, in boys aged 9–14.5 years, we estimated the association between PFAS concentrations and age-adjusted *z*-scores of LH and FSH, and the odds of having a prepubertal testosterone level (<0.5 nmol/L). AORs were estimated using logistic regression, while continuous hormone outcomes (*z*-scores of LH and FSH) were analyzed with linear regression.

Potential confounders and colliders were identified with a directed acyclic graph (DAG) prior to the analysis (Figure S1). Breastfeeding duration and parents’ educational level were identified as factors that may influence pubertal timing^33,34^ and PFAS levels,^3,35^ but they were not included in the main analyses as this would substantially reduce the analysis sample size due to limited responses for these items in the questionnaires. However, these variables were included in a sensitivity analysis. We did not adjust for BMI in our main analyses because it was identified as a possible collider since hormonal changes during puberty lead to an increase in BMI,^36,37^ and PFAS exposure may affect BMI.^38^ However, to assess the possible impact of BMI, BMI *z*-score was included in a sensitivity analysis. Dietary questionnaire data were inadequate for the analysis due to incomplete information regarding the consumption of food groups that contribute substantially to PFAS exposure such as fish, meat, and dairy products.

Serum concentrations of six PFAS (PFOS, PFOA, PFNA, PFHxS, PFDA and PFUnDA) were standardized using robust scaling by subtracting the mean from the serum concentration and dividing the difference by the interquartile range. PFHpS and PFHpA, which both had a considerable proportion of samples below the limit of quantification (LOQ) (Table 2) were dichotomized as being either below or above the LOQ. Eleven PFAS were not included in the statistical analyses as more than 90% of the children had levels below the LOQ.

**Table 2:** Age-Adjusted Logistic Regression Analysis of Having a Prepubertal Testicular Volume (USTV < 2.7 mL, n = 228), a Testicular Volume <7.2 mL (*n* = 193), and a Testicular Volume <17.6 mL (*n* = 150) in Relation to PFAS Concentrations in Boys in the Bergen Growth Study 2 (2016, Norway)^a^

		**USTV < 2.7** **mL**		**USTV < 7.2 mL**		**USTV < 17.6 mL**	
	%>LOQ	AOR (95% CI)	*p* value	AOR (95% CI)	*p* value	AOR (95% CI)	*p* value
PFOS	100	1.82 (1.15, 3.08)	0.016^b^	0.88 (0.53, 1.49)	0.625	2.27 (0.93, 6.52)	0.095
PFOA	100	1.25 (0.75, 2.19)	0.406	1.27 (0.67, 2.42)	0.466	1.15 (0.52, 2.70)	0.730
PFNA	100	1.50 (1.00, 2.32)	0.058	0.89 (0.57, 1.43)	0.618	1.06 (0.53, 2.18)	0.871
PFHxS	100	1.26 (0.99, 1.70)	0.071	1.56 (1.19, 2.26)	0.004^b^	1.25 (0.89, 2.41)	0.333
PFDA	99–100	1.82 (1.05, 3.29)	0.039^b^	1.26 (0.69, 2.31)	0.452	1.91 (0.84, 5.13)	0.149
PFUnDA	84–90	1.88 (1.15, 3.23)	0.016^b^	0.97 (0.57, 1.63)	0.897	1.60 (0.80, 3.85)	0.225
PFHpS	51–66	1.42 (0.61, 3.33)	0.416	0.23 (0.08, 0.59)	0.004^b^	0.85 (0.30, 2.41)	0.758
PFHpA	22–25	0.73 (0.28, 1.93)	0.524	0.74 (0.28, 1.95)	0.543	0.27 (0.08, 0.90)	0.033^b^
∑4PFAS	100	2.20 (1.29, 3.93)	0.005^b^	1.12 (0.64, 2.00)	0.700	1.84 (0.82, 4.65)	0.160
potency score	100	2.20 (1.30, 3.96)	0.005^b^	0.90 (0.52, 1.58)	0.713	1.77 (0.78, 4.45)	0.191

a USTV < 2.7 mL = ultrasound-measured testicular volume less than 2.7 mL; USTV < 7.2 mL = ultrasound-measured testicular volume less than 7.2 mL; USTV < 17.6 mL = ultrasound-measured testicular volume less than 17.6 mL; LOQ = limit of quantification (0.05 ng/mL); AOR = age-adjusted odds ratio; CI = confidence interval; ∑4PFAS = sum of PFOS, PFOA, PFNA, PFHxS; Potency score = the sum of PFOS, PFOA, PFNA and PFHxS, weighted by potency factors of 3, 1, 5, and 0.6, respectively. Age limits: 9–14.5 years for USTV < 2.7 mL, 11–16 years for USTV < 7.2 mL, and 12–16 years for USTV < 17.6 mL. PFOS, PFOA, PFNA, PFHxS, PFDA, PFUnDA, ∑4PFAS and the potency score were standardized using robust scaling with interquartile range. PFHpS and PFHpA concentrations were categorized as either below or above the quantification limit.

b Statistically significant p-values defined at a 0.05-level.

In addition to the implicit adjustment for age by using *z*-scores, age was included in the analyses to account for both the duration and variability of PFAS exposure throughout the children’s lives. This approach considers the variations in serum concentrations over recent decades^39^ attributable to shifts in PFAS production, as well as for the change in PFAS levels during childhood resulting from factors such as growth dilution^40,41^ and alterations in calorie intake per kilogram of body weight.^42^

We modeled each PFAS in single-pollutant models and then used Bayesian and elastic net regression to obtain estimates where each PFAS was adjusted for all the others. ∑4PFAS and potency score were only assessed in the “single pollutant” analyses. Boys with missing data on pubertal markers were excluded from the specific analyses (0–2.6% of the participants). We tested for interactions between the significant PFAS in the single-pollutant model by introducing product terms between them. Additive interactions were evaluated using the Relative Excess Risk due to Interaction (RERI). RERI was calculated for each significant interaction term to quantify the excess risk attributable to the interaction beyond the sum of the individual effects.

Cumulative incidence curves for achieving a pubertal testicular volume (USTV ≥ 2.7 mL) in the three distinct ∑4PFAS tertile groups were generated using a generalized linear model with a binary outcome and a logit link function in the boys aged 9–14.5 years. The corresponding mean (SD) age of reaching puberty across different ∑4PFAS tertiles was calculated using a similar model with a probit link. Descriptive statistics, elastic net, Bayesian, and logistic regression models were analyzed in R version 4.2.3 (R foundation for Statistical Computing).

### Ethical Considerations

The study was approved by the Norwegian Regional Committee for Medical and Health Research Ethics West (reference number 2015/128). A signed informed consent was obtained from a parent or legal guardian of the participating child, and from participants 12 years of age and older. All children received age-appropriate information ahead of examination, and subsequent verbal assent was a requirement. A cinema voucher was given as an incentive to participate in the study.

## Results

Among the 228 boys included in the analyses of pubertal onset, 131 (57%) had an USTV less than 2.7 mL, categorizing them as prepubertal. Out of these, nine boys exhibited a serum testosterone level of ≥0.5 nmol/L despite their low USTV, and 19 had reached Tanner stage PH2. The earliest age of puberty onset was 9.8 years. One boy above the age of 14.5 years was still categorized as prepubertal based on USTV. There were only minor differences in the proportion of missing values between the prepubertal and pubertal boys (Table 1).

The distribution of PFAS concentrations in serum samples from the 300 boys above nine years of age is shown in Table S1. PFOS and PFOA had the highest serum concentrations with a geometric mean of 2.79 and 1.35 ng/mL, respectively, in all boys above nine years of age. Generally, the proportion of PFAS above LOQ and the geometric mean concentrations were higher in boys included in the pubertal onset analyses (9–14.5 years) compared to those included in the analyses on midpubertal and near mature pubertal markers (11–16 and 12–16 years) (Table S2). PFOS, PFOA, PFHxS and PFNA were present in all samples, while PFDA was detected in 99–100%, PFUnDA in 84–90%, PFHpS in 51–66% and PFHpA in 21–24% of the samples in the three age groups. There was a significant positive correlation between all PFAS (Spearman’s correlation coefficient 0.21–0.83) except PFHpA which had a weak significant positive correlation with PFOA, PFNA and PFUnDA (correlation coefficient 0.12–0.23), and a weak nonsignificant positive correlation with PFOS, PFHxS, PFDA and PFHpS. The strongest correlations were between PFDA and PFUnDA (correlation coefficient 0.83) and PFOS and PFDA (correlation coefficient 0.70) (Figure S2).

### Testicular Volume

In the pubertal onset analysis, logistic regression of single pollutants showed that boys with higher levels of all PFAS except PFHpA had a higher odds of being prepubertal (USTV < 2.7), significant for PFOS, PFDA and PFUnDA (Table 2). Higher levels of ∑4PFAS and potency score were also significantly associated with later pubertal onset by USTV. Sensitivity analyses showed that additional adjustment for BMI, breastfeeding duration, and parents’ educational level had minimal impact on the estimates (Table S3). In the interaction analysis, PFNA appeared to have a positive interaction with PFHxS in the association with pubertal onset by USTV (p-interaction = 0.054, RERI = 0.91).

**Figure 2:**
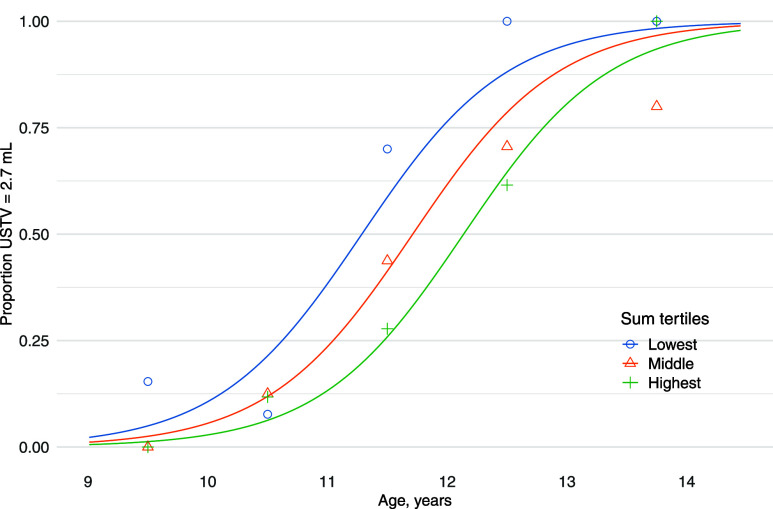
Proportion of boys having attained a pubertal testicular volume in each ∑4PFAS tertile group in boys aged 9–14.5 years in the Bergen Growth Study 2 (2016, Norway) (*n* = 228) USTV ≥ 2.7 mL = ultrasound-measured pubertal testicular volume of ≥2.7 mL. A generalized linear model was used to estimate the cumulative distribution curve in each tertile group of the sum of PFOS, PFOA, PFNA and PFHxS (∑4PFAS). The mean ages of reaching a pubertal testicular volume in the lowest, middle, and highest tertile were calculated as 11.26, 11.70, and 12.14 years.

We found later pubertal onset with increasing levels of ∑4PFAS using cumulative incidence curves (Figure 2). The mean age at which a pubertal testicular volume was reached was 11.26 years for boys in the lowest ∑4PFAS tertile, 11.70 years for those in the middle tertile, and 12.14 years for those in the highest tertile.

**Figure 3:**
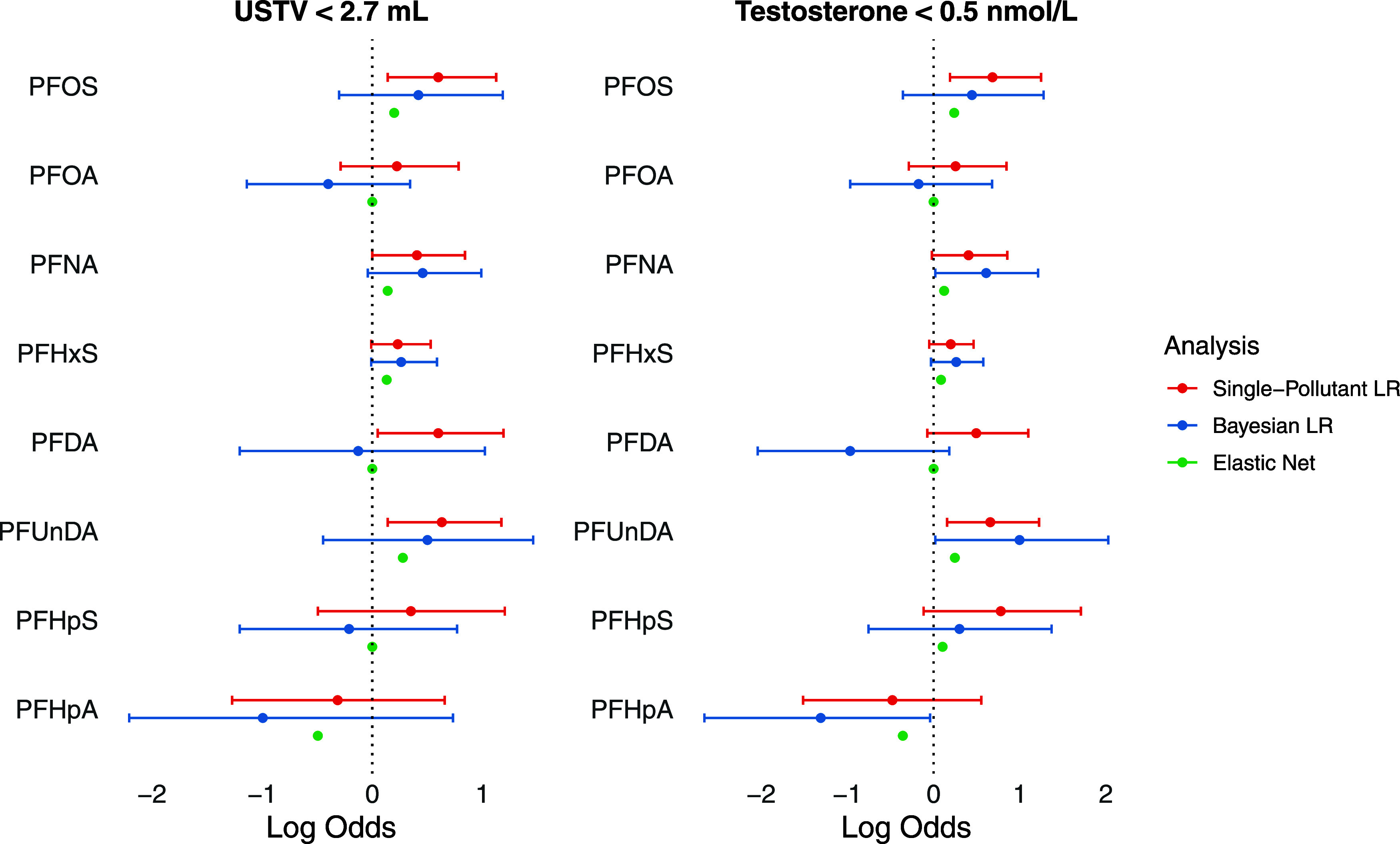
Associations between PFAS serum concentrations and being prepubertal based on ultrasound-measured testicular volume (USTV) (*n* = 224) and serum testosterone level (*n* = 226) in boys aged 9–14.5 years in the Bergen Growth Study 2 (2016, Norway) LR = Logistic Regression. Log odds (with 95% confidence intervals/credible intervals) represent the log odds of being prepubertal and are adjusted for age in all analyses, and for the other PFAS in the Bayesian logistic regression analysis and elastic net analysis. PFOS, PFOA, PFNA, PFHxS, PFDA and PFUnDA were standardized using robust scaling with interquartile range. PFHpS and PFHpA concentrations were categorized as either below or above the quantification limit of 0.05 ng/mL.

In the Bayesian logistic regression model, higher levels of PFNA and PFHxS were associated with being prepubertal based on USTV, which was also supported by elastic net. In addition, elastic net also selected PFOS and PFUnDA as being associated with higher odds of being prepubertal, while PFHpA was associated with lower odds. The results from the three different models are presented in Figure 3.

In the analyses focusing on more advanced stages of testicular development, the direction of the association was less consistent. Notably, higher levels of PFHxS were associated with having a midpubertal USTV less than 7.2 mL in the single-pollutant analysis (Table 2), the Bayesian logistic regression analysis (Table S4), and the elastic analysis, where PFHxS was the strongest predictor (Table S5). Further, we found that detectable levels of PFHpS were associated with lower odds of USTV < 7.2 mL in all three models (Tables 2, and S4, S5). Similarly, detectable levels of PFHpA were associated with lower odds of USTV < 17.6 mL in the single-pollutant model (Table 2) and remained significant after adjusting for the other PFAS with Bayesian (Table S4) and elastic net modeling (Table S5). No significant associations were found between the other PFAS and these outcomes.

### Tanner PH Stages

Boys with higher PFOS levels had significantly higher odds of being in a prepubarche stage (Tanner PH < 2) in the single-pollutant analysis (Table 3), and this was supported by Bayesian logistic regression (Table S6) and elastic net analysis (Table S5). No significant associations were found between PFAS levels and having a Tanner PH < 5 (Table 3).

**Table 3:** Age-Adjusted Logistic Regression Analysis of Having a Tanner Pubic Hair Stage Less than 2 (*n* = 222) and a Tanner Pubic Hair Stage Less than 5 (*n* = 150), in Relation to PFAS Concentrations in Boys in the Bergen Growth Study 2 (2016, Norway)^a^

		**Tanner PH < 2**		**Tanner PH < 5**	
	%>LOQ	AOR (95% CI)	*p* value	AOR (95% CI)	*p* value
PFOS	100	1.67 (1.06, 2.77)	0.036^b^	1.08 (0.57, 2.11)	0.809
PFOA	100	1.21 (0.72, 2.10)	0.489	1.45 (0.73, 2.89)	0.287
PFNA	100	0.81 (0.57, 1.18)	0.251	1.73 (0.94, 3.30)	0.087
PFHxS	100	1.24 (0.98, 1.66)	0.094	1.04 (0.76, 1.41)	0.835
PFDA	99–100	1.15 (0.68, 1.99)	0.600	1.83 (0.94, 3.68)	0.079
PFUnDA	84–90	1.14 (0.74, 1.79)	0.568	1.29 (0.73, 2.28)	0.372
PFHpS	51–66	1.38 (0.59, 3.24)	0.450	1.42 (0.57, 3.52)	0.449
PFHpA	22–25	1.05 (0.40, 2.79)	0.929	0.56 (0.19, 1.58)	0.281
∑4PFAS	100	1.62 (0.98, 2.77)	0.067	1.31 (0.68, 2.58)	0.422
potency score	100	1.40 (0.87, 2.33)	0.180	1.38 (0.71, 2.74)	0.349

a Tanner PH < 2 = Tanner pubic hair stage less than 2; Tanner PH < 5 = Tanner pubic hair stage less than 5; LOQ = limit of quantification (0.05 ng/mL); AOR = age-adjusted odds ratio; CI = confidence interval; ∑4PFAS = sum of PFOS, PFOA, PFNA, PFHxS; Potency score = the sum of PFOS, PFOA, PFNA and PFHxS, weighted by potency factors of 3, 1, 5 and 0.6, respectively. PFOS, PFOA, PFNA, PFHxS, PFDA, PFUnDA, ∑4PFAS and the potency score were standardized using robust scaling with interquartile range. PFHpS concentrations were categorized as either below or above the quantification limit. For Tanner PH2, boys between 9 and 14.5 years of age were included, and boys 12–16 years were included for Tanner PH 5. The referent group was Tanner PH ≥ 2 for boys 9–14.5 years and Tanner PH 5 for boys aged 12–16 years old.

b Statistically significant p-value defined at a 0.05-level.

### Hormone Levels

Single-pollutant analysis showed that boys with higher levels of all PFAS except PFHpA had lower LH *z*-scores and higher odds of prepubertal testosterone levels (<0.5 nmol/L), significant for PFOS, PFUnDA, ∑4PFAS and potency score (Table 4). Higher levels of ∑4PFAS and potency score were also significantly associated with lower LH *z*-scores and prepubertal testosterone levels. When adjusting for the other PFAS with Bayesian modeling, all credible intervals contained 0 for LH *z*-scores, while there was a positive association between PFNA and PFUnDA and prepubertal testosterone levels (Table S7). Elastic net analysis showed a negative association between PFOS and LH *z*-scores, and a positive association between PFOS, PFNA and PFUnDA and prepubertal testosterone levels. Detectable levels of PFHpA were associated with lower odds of prepubertal testosterone levels in both the Bayesian and elastic net model (Tables S7, S8). The log odds of prepubertal testosterone levels from the three different models are shown in Figure 3.

**Table 4:** Age-Adjusted Linear Regression for Z-Scores of LH (*n* = 224) and FSH (*n* = 226) and Age-Adjusted Logistic Regression for Having a Serum Testosterone <0.5 nmol/L (*n* = 226), in Relation to PFAS Concentrations in Boys Aged 9–14.5 Years in the Bergen Growth Study 2 (2016, Norway)^a^

		**LH *z*-score**		**FSH *z*-score**		**serum testosterone <0.5 nmol/L**	
	%>LOQ	estimate (SE)	*p* value	estimate (SE)	*p* value	AOR (95% CI)	*p* value
PFOS	100	–0.20 (0.07)	0.006^b^	–0.10 (0.07)	0.177	1.98 (1.21, 3.48)	0.011^b^
PFOA	100	–0.05 (0.08)	0.555	–0.03 (0.08)	0.733	1.29 (0.75, 2.33)	0.380
PFNA	100	–0.07 (0.06)	0.247	–0.08 (0.06)	0.194	1.50 (0.98, 2.35)	0.066
PFHxS	100	–0.05 (0.04)	0.212	–0.02 (0.04)	0.683	1.22 (0.95, 1.59)	0.120
PFDA	100	–0.09 (0.09)	0.317	–0.11 (0.09)	0.204	1.64 (0.93, 3.00)	0.092
PFUnDA	90	–0.16 (0.08)	0.037^b^	–0.12 (0.08)	0.111	1.93 (1.17, 3.40)	0.015^b^
PFHpS	65	–0.23 (0.14)	0.105	0.06 (0.14)	0.667	2.18 (0.89, 5.52)	0.093
PFHpA	21	0.14 (0.17)	0.414	–0.09 (0.16)	0.583	0.62 (0.22, 1.74)	0.360
∑4PFAS	100	–0.21 (0.08)	0.009^b^	–0.12 (0.08)	0.152	2.35 (1.34, 4.36)	0.004^b^
potency score	100	–0.22 (0.08)	0.007^b^	–0.13 (0.08)	0.101	2.39 (1.37, 4.50)	0.004^b^

a LH = luteinizing hormone; FSH = follicle-stimulating hormone; LOQ = limit of quantification (0.05 ng/mL); SE = standard error; AOR = age-adjusted odds ratio; CI = confidence interval; ∑4PFAS = sum of PFOS, PFOA, PFNA, PFHxS; Potency score = the sum of PFOS, PFOA, PFNA and PFHxS, weighted by potency factors of 3, 1, 5, and 0.6, respectively. In both models, PFOS, PFOA, PFNA, PFHxS, PFDA, PFUnDA, ∑4PFAS and the potency score were standardized using robust scaling with interquartile range. PFHpS and PFHpA concentrations were categorized as either below or above the quantification limit.

b Statistically significant p-values defined at a 0.05-level.

In the interaction analysis, PFNA appeared to have a positive interaction with PFHxS (p-interaction = 0.005, RERI = 1.30) and a negative interaction with PFOS (p-interaction = 0.003, RERI = −1.64) in the association with testosterone. No significant associations were observed for FSH.

## Discussion

Utilizing data from BGS2, our study examined associations between PFAS exposure and pubertal timing in 300 boys living in Bergen, Norway. We used ultrasound-measured testicular volume as an objective evaluation of pubertal status, complemented by assessments of Tanner PH stages and hormone levels, thus addressing a notable gap in the existing literature. Our findings indicate that serum concentrations of certain PFAS were associated with later attainment of distinct pubertal markers. Particularly, higher levels of ∑4PFAS and potency score were associated with later pubertal onset by testicular volume, while higher levels of PFOS corresponded to later pubarche by Tanner staging. Furthermore, higher levels of ∑4PFAS and potency score were associated with lower LH *z*-scores and later pubertal onset based on testosterone levels. In general, the direction of associations was the same between the different measures of pubertal onset, strengthening these findings.

We focused on a healthy group of boys exhibiting a range of normal pubertal development whose PFAS levels were quite similar to other individuals with background exposure levels.^3,7^ Geometric mean PFAS concentrations in children above 12 years of age (boys and girls) in the BGS2 were comparable to those in children included in the European Human Biomonitoring Initiative (HBM4 EU) aligned studies (aged 12–18 years), where also Norwegian samples were included, i.e., PFOS 2.37 vs 2.13 ng/mL, PFOA 1.20 vs 0.97 ng/mL, PFNA 0.70 vs 0.30 ng/mL and PFHxS 0.42 vs 0.41 ng/mL.^6,27^

Most of the previous studies have examined associations between exposure to one PFAS at a time and different health effects, thereby not adjusting for the correlation between the different PFAS. To obtain estimates where each PFAS was coadjusted for the other PFAS, we used Bayesian modeling and elastic net in addition to single-pollutant analysis. ∑4PFAS and the potency score are different ways to account for the possible additive effect of these PFAS. For the significant results, we tested for interactions between the individual PFAS. PFNA appeared to have a positive interaction with PFHxS in the association with testosterone and USTV, and a negative interaction with PFOS in the association with testosterone. In vitro studies suggest the potential for synergistic or antagonistic interactions depending on the species, dose level, dose ratio, and mixture component,^43^ and these should be explored in future human studies.

Adjusting for BMI, breastfeeding duration, and parents’ educational level in sensitivity analyses had minimal impact on the estimates, indicating that differences in these variables do not explain the observed association between PFAS exposure and later pubertal onset.

Interestingly, the results from the analyses using potency score or the unweighted sum of PFOS, PFOA, PFNA and PFHxS were very similar. This suggests that the total exposure to these PFAS is a critical factor to consider when assessing pubertal development, but it is not possible to decide which summing approach is most appropriate. In both approaches the more common PFAS are driving the associations. Further, Bayesian modeling and elastic net showed that PFOS, PFNA and PFHxS, together with PFUnDA, had the strongest correlations with later pubertal onset by USTV and testosterone level, while PFOA showed no association. This could indicate that other potency factors than those available for liver effects in rodents are more relevant when assessing pubertal development.

The observed association between higher PFAS levels and later attainment of a specific testicular volume, was strongest at the onset of puberty. This could be due to greater variability in testicular volume during later stages of puberty (Figure S3). Additionally, the sample sizes for the analyses of midpubertal and adult testicular volumes were somewhat smaller, potentially influencing these findings.

Boys with measurable levels of PFHpS and PFHpA were more likely to have reached a midpubertal and adult testicular volume, respectively. However, significant correlations were not observed with pubertal onset by USTV or Tanner PH. The high proportion of samples below LOQ (75–79% for PFHpA and 34–49% for PFHpS), and the low concentrations in samples above LOQ, implying higher uncertainty in the analyses, might affect the reliability of the observed associations. Further, PFHpA has the shortest half-life among those included in our analyses,^44^ indicating that serum level of PFHpA represents more recent exposure. Children in advanced stages of puberty have a higher calorie intake per kilogram body weight,^45^ and consequently have a higher intake of PFAS. This could explain the observed association between measurable levels of PFHpA and a greater likelihood of having reached a more advanced stage of testicular volume. Finally, different PFAS might have different endocrine-disrupting properties, and future research should look further into the potential effects of exposure to PFAS with shorter half-lives on pubertal development.

A recent cross-sectional study of Norwegian teenagers aged 15–19 years, found that higher levels of certain PFAS were associated with self-reported early menarche in girls, and in contrast to our findings, a more advanced pubertal development in boys.^13^ Further, a prospective cohort study of children in Boston, US, found associations between higher PFAS levels and parent-reported later markers of pubertal timing in girls, but no associations with pubertal timing in boys.^14^ Although existing studies are limited and varied, several rodent and human studies indicate that higher PFAS exposure is associated with later pubertal maturation.^10,12,18^ Our results align with these findings.

Rodent studies have suggested that PFAS may contribute to a delay in puberty by impaired testosterone biosynthesis as reported in rats (PFNA, PFDA) and mice (PFNA).^46,47^ Another suggested mechanism is inhibition of the androgen receptors as reported in vitro at relatively high concentrations.^48^ Further, studies on adult female mice suggest that high levels of PFAS can impact the hypothalamic neurons controlling the hypothalamic-pituitary–gonadal axis.^49^ It is not known to which extent these mechanisms are relevant at the exposure levels in boys in our study group. However, the association between higher PFAS levels and lower LH *z*-scores in the present study suggests a central mechanism for later pubertal onset.

The observed correlations between higher levels of ∑4PFAS or potency scores with lower LH *z*-scores or having a prepubertal serum testosterone level, align with the association between PFAS levels and testicular volume. The relationship can be explained by LH’s role in stimulating testosterone production which subsequently stimulates testicular growth.^50,51^ Additionally, LH may also have an independent effect on testicular growth.^50^ However, the first stimulus for testicular growth in puberty is by FSH stimulating the Sertoli cells, and the clinical onset of puberty by testicular volume is FSH mediated. Given this, we would expect to find associations between PFAS exposure and FSH *z*-scores, which we did not. On the other hand, the major testicle growth during puberty is LH/androgen driven, and this can explain the observed associations with LH and testosterone, but not with FSH. Cross-sectional associations between postnatal PFAS exposure and lower testosterone levels have also been reported in American boys aged 6–9 years^52^ and in Taiwanese boys aged 13–15 years,^53^ though these boys had markedly higher levels of PFAS compared to our sample population.

The cross-sectional design limits our ability to determine causality in the observed associations. Several animal and cohort studies in humans have indicated a causal link between PFAS exposure and later pubertal development in both sexes,^10−12,18^ though published data is still limited and variable. Further, we could not account for the potential impact of the PFAS not included in the analyses or other substances with endocrine-disrupting potential such as polychlorinated biphenyl (PCBs), flame retardants, phthalates, polychlorinated phenols/pesticides^54^ and blood metals^55^ due to lack of available data on these substances. In addition, alcohol and tobacco use are known factors associated with altered testosterone level,^56,57^ but these variables were not included in our questionnaires. Furthermore, a risk of bias amplification is created by the correlations among included PFAS levels and the lack of adjustment for known and other potential unknown confounders in the Bayesian and elastic net models.^58^ This could lead to more skewed estimates than in the single-pollutant analysis, and make it challenging to interpret results and isolate the contribution of each PFAS. This highlights the importance of identification and control of confounders, and the choice of an appropriate study design in future studies.

Among the boys who provided ethnicity data via questionnaires (34%), the majority were of Norwegian origin (75%), while 14% had at least one parent from another European country, and 11% were from a non-European country. The sample closely represents the demographic structure of the Norwegian population in 2016.^59^ This ethnic composition may limit the generalizability of our findings to other populations with different ethnic backgrounds.

Strengths of our study include the use of different measures on pubertal onset and development, using ultrasound measurements of testicular volume, Tanner PH evaluation, and levels of pubertal hormones. Furthermore, we use Bayesian modeling and elastic net analysis to obtain estimates where each PFAS was adjusted for the others to assess the contribution of the individual PFAS. Finally, our analyses of association of a range of PFAS and pubertal timing in a cohort with background exposure, provide valuable insights of relevance for public health.

In conclusion, the present study showed that higher levels of the ∑4PFAS was significantly associated with later pubertal onset in boys, as assessed by ultrasound-measured testicular volume and testosterone level. This was supported by Bayesian and elastic net regression, which showed that higher levels of PFNA and PFHxS were associated with later pubertal onset by USTV, while higher levels of PFNA and PFUnDA were associated with later pubertal onset by testosterone level. In general, the direction of associations was similar across different measures of puberty, strengthening our findings. Further research should include longitudinal studies with repeated measurements of PFAS throughout childhood, along with assessments of prenatal exposure. This design could support causality in the observed association between PFAS exposure and later puberty. Additionally, a deeper understanding of the modes of action is needed, and the combined effects and potential interactions of EDCs mixtures should be further explored.

## Supporting Information Available

Table S1: PFAS serum concentrations; Table S2: Proportion of samples above LOQ and GM (95% CI) in different age groups; Table S3: Sensitivity analyses; Table S4: Bayesian logistic regression analysis, USTV < 2.7 mL, < 7.2 mL and <17.6 mL; Table S5: Elastic net analysis, USTV < 2.7 mL, < 7.2 mL, < 17.6 mL, Tanner PH < 2 and <5; Table S6: Bayesian logistic regression analysis, Tanner PH < 2 and <5; Table S7: Bayesian linear regression analysis for LH and FSH *z*-scores, and Bayesian logistic regression analysis for testosterone; Table S8: Elastic net analysis for LH and FSH *z*-scores, and testosterone; Figure S1: Directed Acyclic Graph; Figure S2: Spearman correlation heatmap; Figure S3: USTV by age (PDF)
