# Supplementary material for: Exposure to Per- and Polyfluoroalkyl Substances and Timing of Puberty in Norwegian Boys: Data from the Bergen Growth Study 2
Source: Environ Sci Technol. 2024 Sep 3;58(37):16336–46. doi: 10.1021/acs.est.4c06062 (PMC11411722; doi:10.1021/acs.est.4c06062)
Supplement: Supplementary file 1 — es4c06062_si_001.pdf [file es4c06062_si_001.pdf]

Supporting Information:

Exposure to Per- and Polyfluoroalkyl Substances and Timing of Puberty in Norwegian Boys: Data from the Bergen Growth Study 2

Ingvild Halsør Forthun [a,b], Mathieu Roelants [c], Helle Katrine Knutsen [d,e], Line Småstuen Haug [d,e], Nina Iszatt [d,e], Lawrence M. Schell [f], Astanand Jugessur [g,h], Robert Bjerknes [a,b], Ninnie B. Oehme [b], Andre Madsen [i], Ingvild Særvold Bruserud [b], Petur Benedikt Juliusson [a,b,j]

[a] *Department of Clinical Science, University of Bergen, 5020 Bergen, Norway.*
[b] *Children and Youth Clinic, Haukeland University Hospital, 5021 Bergen, Norway.*
[c] *Department of Public Health and Primary Care, Centre for Environment and Health KU Leuven, 3000 Leuven, Belgium.*
[d] Department of Food Safety, *Norwegian Institute of Public Health, 0213 Oslo, Norway.*
[e] *Center for Sustainable Diets, Norwegian Institute of Public Health, 0213 Oslo, Norway.*
[f] *Department of Epidemiology and Biostatistics, University at Albany, Albany, New York 12144, USA.*
[g] *Centre for Fertility and Health, Norwegian Institute of Public Health, 0213 Oslo, Norway.*
[h] *Department of Global Public Health and Primary Care, University of Bergen, 5020 Bergen, Norway.*
[i] *Medical Biochemistry and Pharmacology, Haukeland University Hospital, 5021 Bergen, Norway.*
[j] *Department of Health Registry Research and Development, Norwegian Institute of Public Health, 5808 Bergen, Norway.*

***Corresponding author**
Ingvild Halsør Forthun, MD
Children and Youth Clinic
Haukeland University Hospital
Bergen, Norway
Phone: +47 91346635
Email: ingvild.halsor.forthun@helse-bergen.no

Number of pages: 14

Number of tables: 8

Number of figures: 3

Table of content:

Table S1: Serum concentrations (ng/mL) of PFAS in 300 boys aged 9-16 years in the Bergen Growth Study 2 (2016, Norway)

Table S2: Proportion of samples above limit of quantification (LOQ) and geometric mean (95% CI) in different age groups in boys in the Bergen Growth Study 2 (2016, Norway)

Table S3: Odds ratios for being prepubertal (USTV < 2.7 mL) in relation to PFAS concentrations, adjusted for (i) age (n = 228), (ii) age and BMI z-score (n = 228), (iii) age (n = 144), (iv) age, breastfeeding duration, and parents' educational level (n = 144) in boys aged 9-14.5 years in the Bergen Growth Study 2 (2016, Norway)

Table S4: Bayesian logistic regression analysis of having an ultrasound testicular volume < 2.7 mL (n = 228), < 7.2 mL (n = 193) and < 17.6 mL (n = 150) in relation to PFAS concentrations, adjusted for age, in boys in the Bergen Growth Study 2 (2016, Norway)

Table S5: Elastic net analysis of having an ultrasound testicular volume < 2.7 mL (n = 228), < 7.2 mL (n = 193), < 17.6 mL (n = 150), a Tanner pubic hair stage < 2 (n = 222) and a Tanner pubic hair stage < 5 (n = 150), in relation to PFAS concentrations, adjusted for age, in boys in the Bergen Growth Study 2 (2016, Norway)

Table S6: Bayesian logistic regression analysis of having a Tanner pubic hair stage < 2 (n = 222) and a Tanner pubic hair stage < 5 (n = 150), in relation to PFAS concentrations, adjusted for age, in boys in the Bergen Growth Study 2 (2016, Norway)

Table S7: Bayesian linear regression analysis for z-scores of LH (n = 224) and FSH (n = 226), and Bayesian logistic regression analysis for serum testosterone < 0.5 nmol/L (n = 226), in relation to PFAS concentrations, adjusted for age, in boys aged 9-14.5 years

Table S8: Elastic net analysis for z-scores of LH (n = 224) and FSH (n = 226), and serum testosterone < 0.5 nmol/L (n = 226), in relation to PFAS concentrations, adjusted for age, in boys aged 9-14.5 years in the Bergen Growth Study 2 (2016, Norway)

Figure S1: Directed Acyclic Graph (DAG), representing the relationship between PFAS levels and pubertal status with possible confounders and colliders

Figure S2: Spearman correlation heatmap between PFAS concentrations in 300 boys aged

9-16 years in the Bergen Growth Study 2 (2016, Norway)

Figure S3: Ultrasound-measured testicular volume (mL) by age in 300 boys aged 9-16 years in the Bergen Growth Study 2 (2016, Norway)

Table S1: Serum concentrations (ng/mL) of PFAS in 300 boys aged 9-16 years in the Bergen Growth Study 2 (2016, Norway)

	LOQ	N>LOQ	%>LOQ	P2.5	P25	P50	P75	P97.5	IQR	Max	AM	GM	95%CI GM
PFOS	0.05	300	100	1.23	2.10	2.77	3.74	6.90	1.64	10.9	3.08	2.79	1.17, 6.62
PFOA	0.05	300	100	0.59	1.09	1.38	1.70	2.50	0.61	3.95	1.44	1.35	0.67, 2.71
PFNA	0.05	300	100	0.28	0.52	0.73	1.00	2.15	0.48	4.48	0.84	0.73	0.26, 2.03
PFHxS	0.05	300	100	0.23	0.41	0.53	0.67	1.63	0.27	4.94	0.62	0.54	0.21, 1.39
PFDA	0.05	298	99	0.06	0.11	0.15	0.20	0.33	0.09	0.51	0.16	0.15	0.06, 0.35
PFUnDA	0.05	261	87	<LOQ	0.07	0.11	0.15	0.30	0.08	0.46	0.12	0.10	<LOQ, 0.33
PFHpS	0.05	182	61	<LOQ	<LOQ	0.06	0.08	0.15	0.05	0.19	0.07	0.06	<LOQ, 0.15
PFHpA	0.05	67	22	<LOQ	<LOQ	<LOQ	<LOQ	0.12	-	0.26	<LOQ	<LOQ	<LOQ, 0.09
PFDoDA	0.05	21	7	<LOQ	<LOQ	<LOQ	<LOQ	0.09	-	0.23	<LOQ	<LOQ	<LOQ, 0.06
PFTrDA	0.05	13	4	<LOQ	<LOQ	<LOQ	<LOQ	0.05	-	0.13	<LOQ	<LOQ	<LOQ, <LOQ
PFTeDA	0.20	1	0.3	<LOQ	<LOQ	<LOQ	<LOQ	<LOQ	-	0.20	<LOQ	<LOQ	<LOQ, <LOQ
∑4PFAS	100	300	100	2.76	4.51	5.63	7.10	11.4	2.60	15.2	5.97	5.61	2.80, 11.3
Potency score	100	300	100	6.90	11.3	14.0	18.4	30.4	7.05	39.4	15.2	14.2	6.74, 29.9

LOQ = limit of quantification; AM = arithmetic mean; GM = geometric mean; 95%CI = 95% confidence interval; IQR = interquartile range; PFOS = perfluorooctanesulfonic acid; PFOA = perfluorooctanoic acid; PFNA = perfluorononaoic acid; PFHxS = perfluorohexanesulfonic acid; PFDA = perfluorodecanoic acid; PFUnDA = perfluoroundecanoic acid; PFHpS = perfluoroheptanesulfonic acid; PFHpA = perfluoroheptanoic acid; PFDoDA = perfluorododecanoic acid; PFTrDA = perfluorotridecanoic acid; PFTeDA = perfluorotetradecanoic acid. ∑4PFAS = sum of PFOS, PFOA, PFNA and PFHxS. Potency score = the sum of PFOS, PFOA, PFNA and PFHxS, weighted by potency factors of 3, 1, 5 and 0.6, respectively. Perfluorobutanoic acid (PFBA), perfluorohexanoic acid (PFHxA), perfluorobutanesulfonic acid (PFBS), perfluorordecanesulfonic acid (PFDS), perfluorooctanesulfonamide (PFOSA), N-methylperfluorooctanesulfonamide (MeFOSA) and N-ethylperfluorooctanesulfonamide (EtFOSA) were below the LOQ in all children. Perfluoropentanoic acid (PFPeA) was detected in a small number of samples, but these measurements are not reported due to reliability concerns flagged by the quality assurance system.

Table S2: Proportion of samples above limit of quantification (LOQ) and geometric mean (95% CI) in different age groups in boys in the Bergen Growth Study 2 (2016, Norway)

	**Pubertal onset 9-14.5 years (n = 228)**		**Mid-puberty 11-16 years (n = 197)**		**Near mature 12-16 years (n = 152)**	
	%>LOQ	GM (95% CI)	%>LOQ	GM (95% CI)	%>LOQ	GM (95% CI)
PFOS	100	2.95 (1.23, 7.07)	100	2.67 (1.17, 6.12)	100	2.55 (1.15, 5.66)
PFOA	100	1.44 (0.75, 2.78)	100	1.25 (0.65, 2.43)	100	1.21 (0.62, 2.39)
PFNA	100	0.76 (0.27, 2.12)	100	0.74 (0.27, 1.98)	100	0.74 (0.27, 2.03)
PFHxS	100	0.58 (0.23, 1.44)	100	0.52 (0.20, 1.36)	100	0.50 (0.19, 1.31)
PFDA	100	0.16 (0.07, 0.36)	99	0.14 (0.06, 0.33)	99	0.13 (0.05, 0.30)
PFUnDA	90	0.11 (<LOQ, 0.35)	85	0.10 (<LOQ, 0.32)	84	0.09 (<LOQ, 0.29)
PFHpS	66	0.06 (<LOQ, 0.15)	56	0.06 (<LOQ, 0.15)	51	0.05 (<LOQ, 0.14)
PFHpA	21	<LOQ (<LOQ, 0.09)	23	<LOQ (<LOQ, 0.09)	24	<LOQ (<LOQ, 0.09)
∑4PFAS	100	5.97 (3.04, 11.7)	100	5.36 (2.73, 10.5)	100	5.17 (2.62, 10.2)
Potency score	100	15.0 (7.22, 31.3)	100	13.7 (6.70, 28.0)	100	13.3 (6.48, 27.2)

LOQ = limit of quantification (0.05 ng/mL); GM = geometric mean; 95%CI = 95% confidence interval; ∑4PFAS = sum of PFOS, PFOA, PFNA and PFHxS. Potency score = the sum of PFOS, PFOA, PFNA and PFHxS, weighted by potency factors of 3, 1, 5 and 0.6, respectively.

Table S3: **Odds ratios for being prepubertal (USTV < 2.7 mL) in relation to PFAS concentrations, adjusted for (i) age (n = 228), (ii) age and BMI z-score (n = 228), (iii) age (n = 144), (iv) age, breastfeeding duration, and parents' educational level (n = 144) in boys aged 9-14.5 years in the Bergen Growth Study 2 (2016, Norway)**

		**(i) Age (n = 228)**		**(ii) Age and BMI z-score (n = 228)**		**(iii) Age (n = 144)**		**(iv) Age, breastfeeding, and education (n = 144)**	
	%>LOQ	AOR (95% CI)	p value	AOR (95% CI)	p value	AOR (95% CI)	p value	AOR (95% CI)	p value
PFOS	100	1.82 (1.15, 3.08)	0.016*	1.87 (1.17, 3.13)	0.012*	1.49 (0.79, 3.04)	0.245	1.63 (0.97, 3.09)	0.092
PFOA	100	1.25 (0.75, 2.19)	0.406	1.18 (0.70, 2.07)	0.557	1.45 (0.71, 3.26)	0.334	1.10 (0.55, 2.29)	0.797
PFNA	100	1.50 (1.00, 2.32)	0.058	1.49 (0.98, 2.35)	0.071	1.52 (0.89, 2.74)	0.142	1.53 (0.90, 2.68)	0.116
PFHxS	100	1.26 (0.99, 1.70)	0.071	1.18 (0.93, 1.58)	0.200	1.04 (0.61, 1.71)	0.890	1.05 (0.73, 1.57)	0.799
PFDA	100	1.82 (1.05, 3.29)	0.039*	2.00 (1.10, 3.75)	0.025*	2.06 (0.96, 4.74)	0.073	1.62 (0.85, 3.30)	0.158
PFUnDA	90	1.88 (1.15, 3.23)	0.016*	2.02 (1.21, 3.54)	0.009*	1.91 (1.00, 3.93)	0.062	1.56 (0.92, 2.91)	0.125
PFHpS	66-72	1.42 (0.61, 3.33)	0.416	1.22 (0.51, 2.92)	0.645	1.93 (0.59, 6.53)	0.275	2.40 (0.75, 8.05)	0.145
PFHpA	22-24	0.73 (0.28, 1.93)	0.524	0.68 (0.25, 1.86)	0.454	0.42 (0.10, 1.62)	0.214	0.64 (0.18, 2.23)	0.479
∑4PFAS	100	2.20 (1.29, 3.93)	0.005*	2.12 (1.24, 3.82)	0.008*	1.77 (0.85, 4.03)	0.145	1.86 (0.98, 3.93)	0.076
Potency score	100	2.20 (1.30, 3.96)	0.005*	2.22 (1.30, 3.99)	0.005*	1.82 (0.91, 4.02)	0.112	1.90 (1.04, 3.92)	0.054

USTV < 2.7 mL = prepubertal testicular volume, less than 2.7 mL (ultrasound); LOQ = limit of quantification (0.05 ng/mL); AOR = age-adjusted odds ratio; CI = confidence interval; ∑4PFAS = sum of PFOS, PFOA, PFNA, PFHxS; Potency score = the sum of PFOS, PFOA, PFNA and PFHxS, weighted by potency factors of 3, 1, 5 and 0.6, respectively. PFOS, PFOA, PFNA, PFHxS, PFDA, PFUnDA, ∑4PFAS and the potency score were standardized using robust scaling with interquartile range. PFHpS and PFHpA concentrations were categorized as either below or above the quantification limit. In analysis (iii), 144 out of 228 boys were randomly selected to be included in the analysis. *Statistically significant p-values defined at a 0.05-level.

Table S4: **Bayesian logistic regression analysis of having an ultrasound testicular volume < 2.7 mL (n = 228), < 7.2 mL (n = 193) and < 17.6 mL (n = 150) in relation to PFAS concentrations, adjusted for age, in boys in the Bergen Growth Study 2 (2016, Norway)**

		**USTV < 2.7 mL**	**USTV < 7.2 mL**	**USTV < 17.6 mL**
	%>LOQ	AOR (95% CI)	AOR (95% CI)	AOR (95% CI)
PFOS	100	1.44 (0.68, 3.10)	0.63 (0.26, 1.51)	3.44 (0.76, 17.4)
PFOA	100	0.72 (0.33, 1.60)	1.55 (0.53, 4.60)	1.60 (0.42, 6.26)
PFNA	100	1.79 (1.05, 3.24)*	0.93 (0.52, 1.71)	0.73 (0.27, 1.98)
PFHxS	100	1.35 (1.02, 1.83)*	2.28 (1.44, 3.96)*	1.18 (0.71, 2.38)
PFDA	99-100	0.71 (0.24, 2.25)	2.05 (0.53, 8.28)	1.55 (0.24, 9.85)
PFUnDA	84-90	1.97 (0.72, 5.26)	0.91 (0.29, 3.08)	1.02 (0.26, 4.11)
PFHpS	51-66	0.77 (0.29, 2.08)	0.07 (0.02, 0.28)*	0.26 (0.06, 1.20)
PFHpA	22-25	0.37 (0.11, 1.23)	0.63 (0.18, 2.23)	0.12 (0.04, 0.31)*

USTV < 2.7 mL = ultrasound-measured testicular volume < 2.7 mL; USTV < 7.2 mL = ultrasound-measured testicular volume < 7.2 mL; USTV < 17.6 mL = ultrasound-measured testicular volume < 17.6 mL; LOQ = limit of quantification (0.05 ng/mL); AOR = adjusted odds ratio; CI = credible interval. Age limits: 9-14.5 years for USTV < 2.7, 11-16 years for USTV < 7.2 mL, and 12-16 years for USTV < 17.6 mL. PFOS, PFOA, PFNA, PFHxS, PFDA and PFUnDA were standardized using robust scaling with interquartile range. PFHpS and PFHpA concentrations were categorized as either below or above the quantification limit. *95% CI not including 1.

Table S5: Elastic net analysis of having an ultrasound testicular volume < 2.7 mL (n = 228), < 7.2 mL (n = 193), < 17.6 mL (n = 150), a Tanner pubic hair stage < 2 (n = 222) and a Tanner pubic hair stage < 5 (n = 150), in relation to PFAS concentrations, adjusted for age, in boys in the Bergen Growth Study 2 (2016, Norway)

		USTV < 2.7 mL	USTV < 7.2 mL	USTV < 17.6 mL	Tanner PH < 2	Tanner PH < 5
	%>LOQ	AOR	AOR	AOR	AOR	AOR
PFOS	100	1.18	0.97	2.80	1.32	1.00
PFOA	100	0.98	1.21	1.30	1.00	1.00
PFNA	100	1.24	1.00	0.88	0.88	1.23
PFHxS	100	1.18	1.60	1.00	1.06	1.00
PFDA	99-100	1.00	1.28	1.28	1.00	1.37
PFUnDA	84-90	1.39	1.00	1.00	1.00	1.00
PFHpS	51-66	1.00	0.18	0.37	1.00	1.00
PFHpA	22-25	0.61	0.86	0.20	1.00	0.76

USTV < 2.7 mL = ultrasound-measured testicular volume < 2.7 mL; USTV < 7.2 mL = ultrasound-measured testicular volume < 7.2 mL; USTV < 17.6 mL = ultrasound-measured testicular volume < 17.6 mL; Tanner PH < 2 = Tanner pubic hair stage < 2; Tanner PH < 5 = Tanner pubic hair stage < 5; LOQ = limit of quantification (0.05 ng/mL); AOR = adjusted odds ratio. Age limits: 9-14.5 years for USTV 2.7 and Tanner PH 2, 11-16 years for USTV 7.2 mL, and 12-16 years for USTV 17.6 mL and Tanner PH 5. The referent group was Tanner PH ≥ 2 for boys 9-14.5 years and Tanner PH 5 for boys aged 12-16 years old. PFOS, PFOA, PFNA, PFHxS, PFDA and PFUnDA were standardized using robust scaling with interquartile range. PFHpS and PFHpA concentrations were categorized as either below or above the quantification limit.

Table S6: Bayesian logistic regression analysis of having a Tanner pubic hair stage < 2 (n = 222) and a Tanner pubic hair stage < 5 (n = 150), in relation to PFAS concentrations, adjusted for age, in boys in the Bergen Growth Study 2 (2016, Norway)

		**Tanner PH < 2**	**Tanner PH < 5**
	%>LOQ	AOR (95% CI)	AOR (95% CI)
PFOS	100	1.97 (0.99, 4.07)	0.58 (0.23, 1.48)
PFOA	100	1.28 (0.62, 2.71)	1.07 (0.39, 3.13)
PFNA	100	0.72 (0.46, 1.17)	1.73 (0.82, 3.82)
PFHxS	100	1.17 (0.90, 1.59)	0.99 (0.69, 1.44)
PFDA	99-100	0.77 (0.26, 2.58)	4.21 (0.93, 20.2)
PFUnDA	84-90	0.93 (0.37, 2.29)	0.58 (0.19, 1.83)
PFHpS	51-66	0.99 (0.37, 2.50)	0.96 (0.29, 3.29)
PFHpA	22-25	1.22 (0.39, 3.81)	0.45 (0.13, 1.59)

Tanner PH < 2 = Tanner pubic hair stage < 2; Tanner PH < 5 = Tanner pubic hair stage < 5; LOQ = limit of quantification (0.05 ng/mL); AOR = adjusted odds ratio; CI = credible interval; PFOS, PFOA, PFNA, PFHxS, PFDA and PFUnDA were standardized using robust scaling with interquartile range. PFHpS and PFHpA concentrations were categorized as either below or above the quantification limit. For Tanner PH2, boys between 9 and 14.5 years of age were included, while boys 12-16 years were included for Tanner PH 5. The referent group was Tanner PH ≥ 2 for boys 9-14.5 years and Tanner PH 5 for boys aged 12-16 years old.

Table S7: Bayesian linear regression analysis for z-scores of LH (n = 224) and FSH (n = 226), and Bayesian logistic regression analysis for serum testosterone < 0.5 nmol/L (n = 226), in relation to PFAS concentrations, adjusted for age, in boys aged 9-14.5 years in the Bergen Growth Study 2 (2016, Norway)

		**LH z-score**	**FSH z-score**	**Testosterone < 0.5 nmol/L**
	%>LOQ	Estimate (95% CI)	Estimate (95%CI)	AOR (95% CI)
PFOS	100	-0.19 (-0.40, 0.03)	-0.08 (-0.30, 0.13)	1.56 (0.70, 3.58)
PFOA	100	-0.01 (-0.23, 0.20)	0.03 (-0.19, 0.25)	0.84 (0.38, 1.97)
PFNA	100	-0.07 (-0.21, 0.07)	-0.06 (-0.20, 0.08)	1.84 (1.02, 3.36)*
PFHxS	100	-0.03 (-0.12, 0.06)	-0.01 (-0.09, 0.08)	1.30 (0.97, 1.78)
PFDA	99	0.30 (-0.03, 0.64)	-0.01 (-0.34, 0.31)	0.38 (0.13, 1.20)
PFUnDA	87	-0.20 (-0.49, 0.07)	-0.06 (-0.34, 0.22)	2.71 (1.02, 7.59)*
PFHpS	61	-0.11 (-0.43, 0.20)	0.18 (-0.13, 0.49)	1.35 (0.47, 3.93)
PFHpA	22	0.28 (-0.07, 0.61)	-0.04 (-0.39, 0.31)	0.27 (0.07, 0.96)*

LH = luteinizing hormone; FSH = follicle-stimulating hormone; LOQ = limit of quantification (0.05 ng/mL); CI = credible interval; AOR = adjusted odds ratio. In both models, PFOS, PFOA, PFNA, PFHxS, PFDA and PFUnDA were standardized using robust scaling with interquartile range. PFHpS and PFHpA concentrations were categorized as either below or above the quantification limit. *95% CI not including 1.

Table S8: Elastic net analysis for z-scores of LH (n = 224) and FSH (n = 226), and serum testosterone < 0.5 nmol/L (n = 226), in relation to PFAS concentrations, adjusted for age, in boys aged 9-14.5 years in the Bergen Growth Study 2 (2016, Norway)

		**LH z-score**	**FSH z-score**	**Testosterone < 0.5 nmol/L**
	%>LOQ	Estimate	Estimate	AOR
PFOS	100	-0.11	0.00	1.27
PFOA	100	0.00	0.00	1.00
PFNA	100	0.00	0.00	1.13
PFHxS	100	0.00	0.00	1.09
PFDA	99	0.00	0.00	1.00
PFUnDA	87	0.00	0.00	1.28
PFHpS	61	0.00	0.00	1.11
PFHpA	22	0.00	0.00	0.70

LH = luteinizing hormone; FSH = follicle-stimulating hormone; LOQ = limit of quantification (0.05 ng/mL); AOR = adjusted odds ratio. PFOS, PFOA, PFNA, PFHxS, PFDA and PFUnDA were standardized using robust scaling with interquartile range. PFHpS and PFHpA concentrations were categorized as either below or above the quantification limit.

Figure S1: Directed Acyclic Graph (DAG), representing the relationship between PFAS levels and pubertal status with possible confounders and colliders

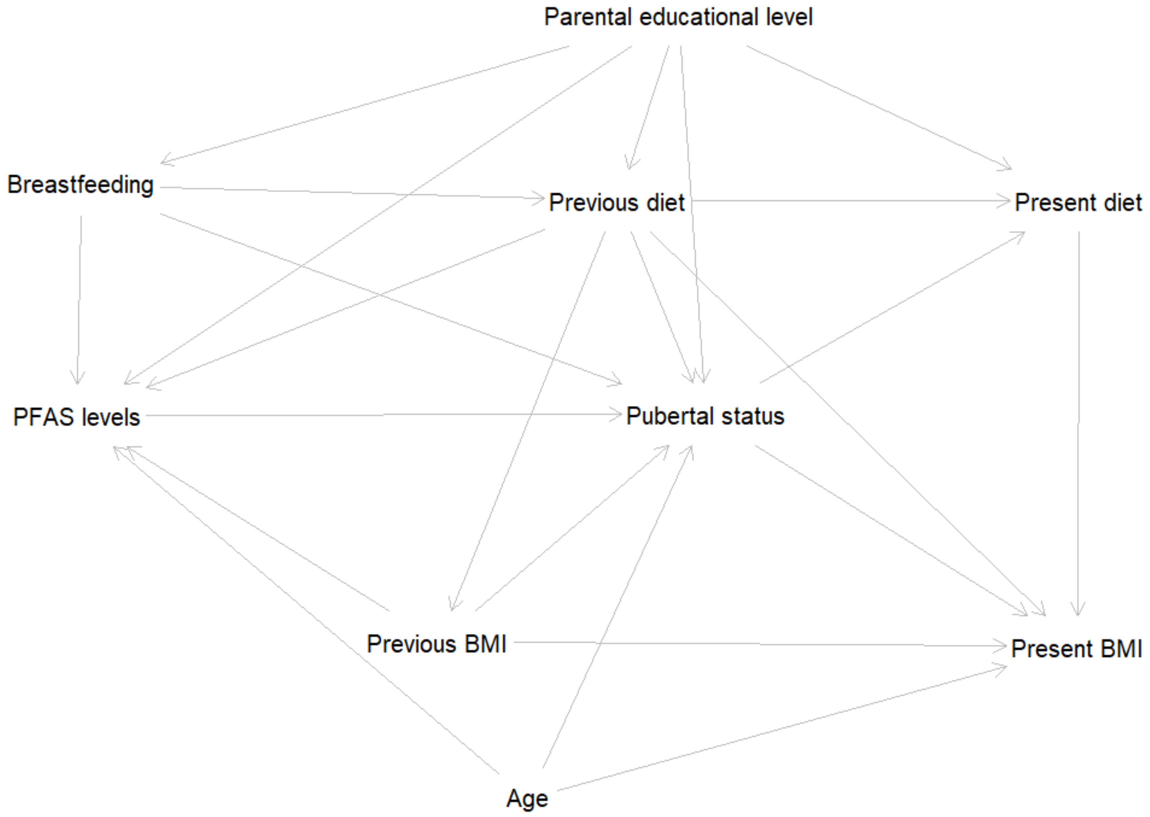

Figure S2: Spearman correlation heatmap between PFAS concentrations in 300 boys aged 9-16 years in the Bergen Growth Study 2 (2016, Norway)

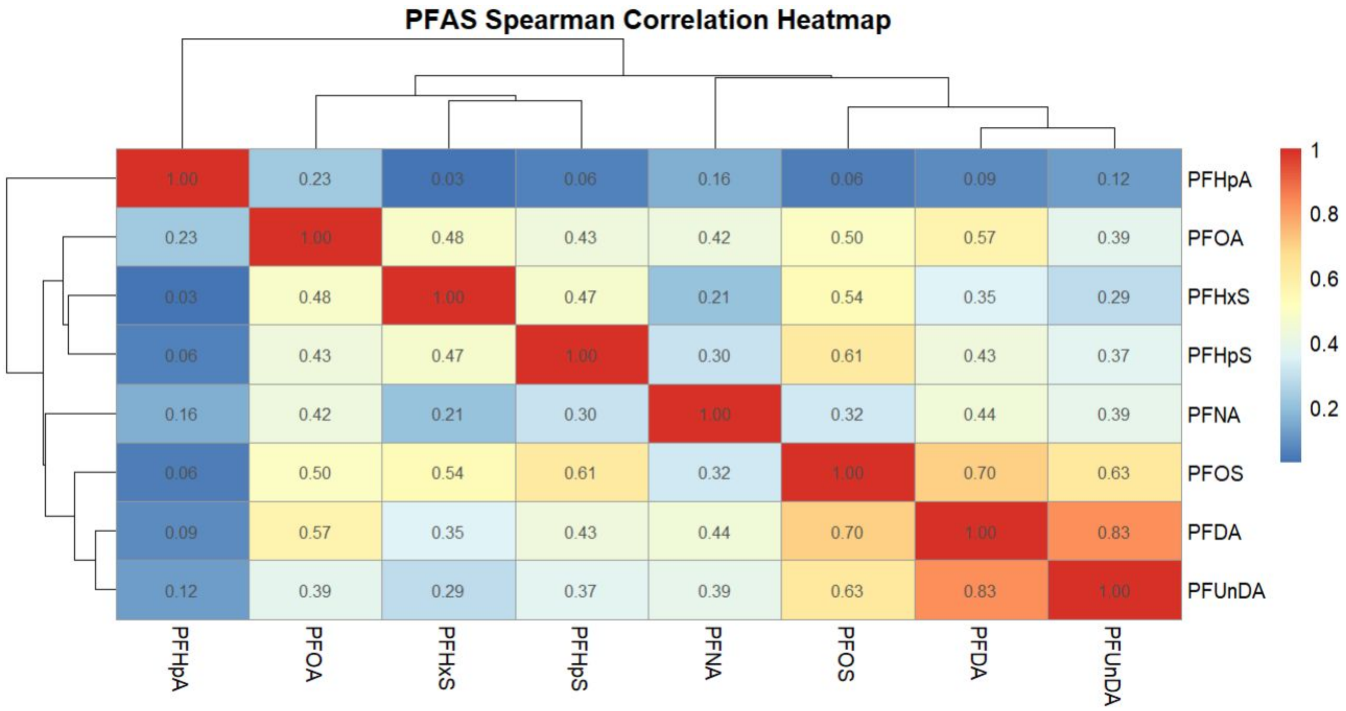

Figure S3: **Ultrasound-measured testicular volume (mL) by age in 300 boys aged 9-16 years in the Bergen Growth Study 2 (2016, Norway)**

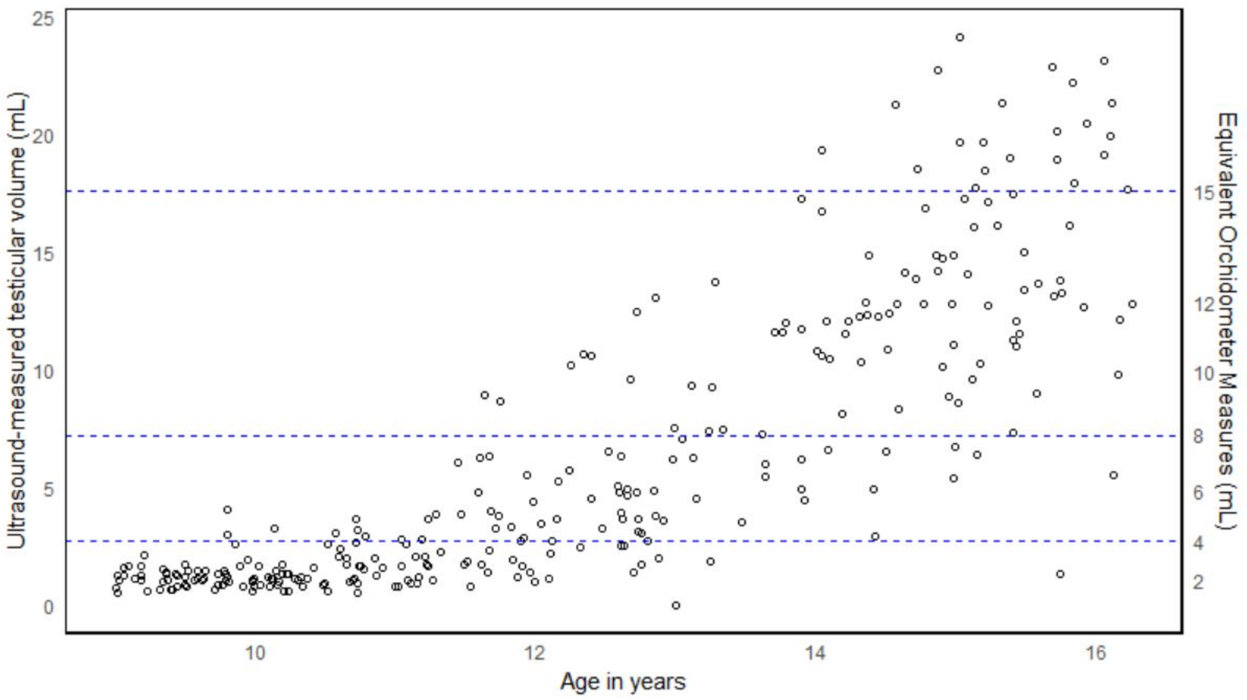

Scatter plot showing ultrasound-measured testicular volume (mL) by age in years, with equivalent orchidometer measures (mL) on the secondary y-axis. The dotted horizontal lines mark ultrasound-measured testicular volume of 2.7 mL (pubertal onset), 7.2 mL (mid-pubertal volume) and 17.6 mL (mature testicular volume).
